# Dietary inflammatory impact on NAFLD development in obese vs. lean individuals: an analysis based on NHANES 2003–2018

**DOI:** 10.1186/s12944-024-02082-4

**Published:** 2024-04-29

**Authors:** Lurao Li, Xiawen Shu, Yun Yi, Chun Wang, Jianghui Li, Yang Ding, Jin Li, Ying Chang

**Affiliations:** 1 Department of Gastroenterology, Zhongnan Hospital of Wuhan University, Wuhan, Hubei 430071 China; 2 Hubei Clinical Center and Key Laboratory of Intestinal and Colorectal Diseases, Wuhan, Hubei China; 3 Department of Laboratory Medicine, Zhongnan Hospital of Wuhan University, Wuhan, Hubei 430071 China

**Keywords:** DII, NAFLD, Lean individuals, Abdominal obesity, BMI, WHtR

## Abstract

**Background:**

Non-alcoholic fatty liver disease (NAFLD), often linked with obesity, can also affect individuals with normal weight, a condition known as “lean NAFLD”, imposing comparable burdens and adverse effects. However, the impact of diet on lean NAFLD remains underexplored. The objective of this study is to investigate the correlation between the Dietary Inflammatory Index (DII) and NAFLD among Americans, stratified by waist-to-height ratio (WHtR) and body mass index (BMI).

**Methods:**

Five thousand one hundred fifty-two participants from the National Health and Nutrition Examination Survey (NHANES) 2003–2018 were comprised in the final analysis. NAFLD and advanced liver fibrosis were diagnosed by serological markers. Lean and abdominal lean individuals were identified using BMI and WHtR, separately. DII was determined by assigning scores to 28 distinct food parameters based on their inflammatory potential, obtained from the NAHNES website. Differences across DII quartiles were evaluated using the Kruskal-Wallis H Test, Chi-Square Test along with One-Way ANOVA. The correlation between DII and NAFLD was determined by multiple regression models and subgroup analyses.

**Results:**

Among the 5152 subjects, 2503 were diagnosed with NAFLD, including 86 cases of lean NAFLD and 8 cases of abdominal lean NAFLD. DII was positively linked with NAFLD (Odds Ratio (OR) = 1.81 [1.48–2.21], *P* < 0.001**)** and advanced liver fibrosis (OR = 1.46 [1.02–2.07], *P* = 0.037). Further analysis revealed that this association was primarily observed in obese or abdominal obese participants (In BMI ≥ 25.00 kg/m^2, OR = 1.56 [1.23–1.98], *P* < 0.001. In WHtR> 0.50, OR = 1.48 [1.23–1.79], *P* < 0.001.), rather than their lean counterparts. Subgroup analyses indicated that female individuals, without a diagnosis of hypertension or diabetes appeared to be more sensitive to the rise in DII.

**Conclusions:**

Our data demonstrated a significant positive correlation between DII and NAFLD in the general population. However, the impact of a pro-inflammatory diet was less prominent in lean individuals compared to obese ones.

**Supplementary Information:**

The online version contains supplementary material available at 10.1186/s12944-024-02082-4.

## Introduction

Non-alcoholic fatty liver disease (NAFLD) is characterized by the accumulation of excess fat in the liver, in the absence of significant alcohol consumption or other long-term liver illnesses, including viral hepatitis or genetic liver disorders [1, 2]. Nowadays, NAFLD is the major cause of end-stage liver disease, primary liver carcinoma and demand for liver transplantation, placing a significant global burden [3]. In addition to its well-established association with obesity, ‘lean NAFLD’ refers to NAFLD affecting individuals with normal weight [4, 5]. The incidence of lean NAFLD varies across regions and races. A meta-analysis revealed that lean NAFLD accounted for approximately 19.20% of NAFLD patients worldwide (95% CI:3.70–7.00) [6]. Previous studies have indicated that lean NAFLD may exhibit comparable outcomes to conventional NAFLD, or potentially even worse liver-related events and overall mortality [7]. To date, genetics, epigenetics, dietary factors, and physical exercise have all been linked to the onset of NAFLD in lean individuals by influencing metabolic flexibility and adaptability [8, 9]. However, the specific mechanisms of lean NAFLD remain unclear. Additionally, specific guidelines for lean NAFLD are absent.

Recent studies have highlighted the critical role of inflammation in the pathogenesis of NAFLD [10, 11]. In humans, the inflammatory balance is maintained by cytokines including IL-6 and IL-1, along with tumor necrosis factor-alpha (TNF-α) and C-reactive protein (CRP) [12]. Disruption of this balance can lead to mild persistent inflammation and tissue damage. Diet plays a pivotal role in modifying the inflammatory state in humans and has been widely used in NAFLD management. Recommendations on NAFLD management include minimizing consumption of a typical Western eating style and advocating for the adoption of a Mediterranean diet. This dietary pattern contains higher intake of omega-3 and monounsaturated fatty acids, lower intake of carbohydrates, refined carbs, and sweets [13–17]. To provide the public with more precise dietary guidance, Shivappa et al. developed the Dietary Inflammatory Index (DII), which is now widely used to quantify the impact of an individual’s diet on inflammation [18]. Previous research has revealed the substantial link between DII and obesity [19], type 2 diabetes (T2DM) [20], hypertension [21] and metabolic dysfunction-associated fatty liver disease (MAFLD) [22]. However, research on the relationship between DII and NAFLD in individuals with diverse weights and body shapes is limited.

Nowadays, several non-invasive serological tests are widely employed in diagnosing NAFLD and advanced liver fibrosis. These tests include the Fatty Liver Index (FLI), the US Fatty Liver Index (USFLI), the Non-Alcoholic Fatty Liver Disease Fibrosis Score (NFS), the Fibrosis 4 Index (FIB-4), the Hepatic Steatosis Index (HSI) and the Aspartate Aminotransferase /Platelet Ratio Index (APRI). Numerous epidemiological studies [23–29] have confirmed the validity of these markers. The objective of this study is to explore associations between DII and NAFLD in different body mass index (BMI) and body shapes (defined by waist-to-height ratio (WHtR)), aiming to provide more detailed dietary advice for NAFLD management.

## Methods

### Population and study design

**Fig. 1:**
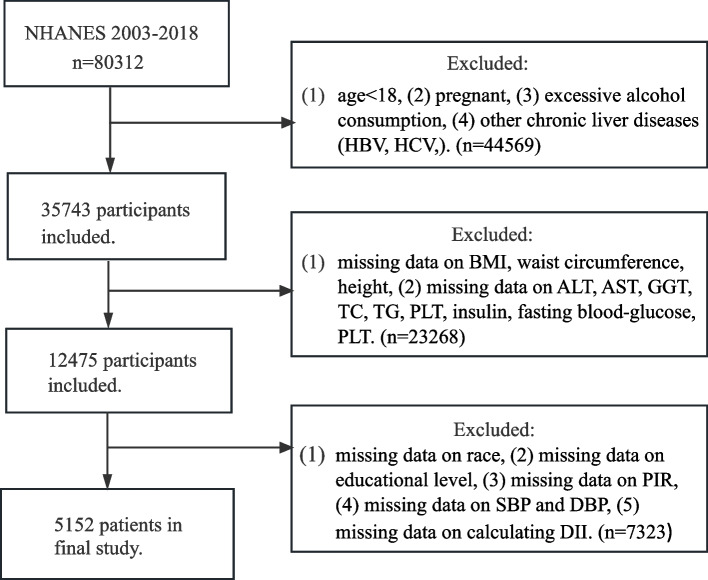
Flow chat of our design

NHANES, a comprehensive database overseen by the Centers for Disease Control and Prevention (CDC), monitors the nutritional status and health of the Americans [30]. Data for this study were obtained from the eight NHANES cycles spanning from 2003 to 2018, as they included all of the relevant variables, readily available on the NHANES official website. The initial dataset consisted of 80,312 individuals. Participants were excluded if they were: (1) under the age of 18, (2) pregnant or were unable to submit a urine sample for testing, (3) had other chronic liver diseases (hepatitis B, C, and liver carcinoma), (4) engaged in excessive alcohol consumption, or (5) had incomplete information on crucial factors, including dietary data, demographic, laboratory and questionnaire. Following this screening procedure, the final research comprised 5152 individuals (Fig. 1 illustrates the study design).

### Diagnostic criteria and definition

#### Definition of NAFLD

As previously stated, NAFLD was defined using FLI and USFLI together. The formulas are shown below:

$$\mathrm{FLI}\;=\;\mathrm e\;\hat{\phantom{0}}\left(0.953\ast\mathrm{In}\;\left(\mathrm{TG}\right)\;+\;0.139\ast\mathrm{BMI}\;+\;0.718\ast\mathrm{In}\left(\mathrm{GGt}\right)\;+\;0.053\ast\mathrm{waist}\;\mathrm{circumference}-15,745\right)/\;\left(1+\mathrm e\hat{\phantom{0}}\left(0.953\ast\mathrm{In}\;(\mathrm{TG})+0.139\ast\mathrm{BMI}+0.718\ast\mathrm{In}\;\left(\mathrm{GGT}\right)\;+\;0.053\ast\mathrm{waist}\;\mathrm{circumference}-15.745\right)\right)\;\ast100.$$

$$\mathrm{USFLI}\;=\;\mathrm e\widehat{\phantom{0}}\left(0.3458\ast\;\mathrm{Mexican}\;\mathrm{American}-0.8073\ast\;\mathrm{non}-\mathrm{Hispanicblack}+\;0.0093\;\mathrm{age}+0.6151\ast\;\mathrm{In}\;\left(\mathrm{GGT}\right)\;+\;0.0249\ast\;\mathrm{waistcircumference}+\;1.1792\ast\;\mathrm{In}\;\left(\mathrm{insulin}\right)\;+\;0.8242\ast\;\mathrm{In}\;\left(\mathrm{Glucose}\right)-\;14.7812\right)/\left(1+\mathrm e\hat{\phantom{0}}\left(0.3458\ast\;\mathrm{Mexican}\;\mathrm{American}-08073\ast\;\mathrm{non}-\mathrm{Hispanicblack}+\;0.0093\ast\;\mathrm{age}+0.6151\ast\;\mathrm{In}\;\left(\mathrm{GGT}\right)\;+\;0.0249\ast\;\mathrm{waitcircumference}+\;1.1792\ast\;\mathrm{In}\;\left(\mathrm{insulin}\right)\;+08242\ast\;\mathrm{In}\;\left(\mathrm{Glucose}\right)\;-\;14.7812\right)\right)\;\ast\;100\;(\left("\mathrm{non}-\mathrm{Hispanic}\;\mathrm{black}"\;\mathrm{and}\;"\mathrm{Mexican}\;\mathrm{American}"\;\mathrm{have}\;\mathrm a\;\mathrm{value}\;\mathrm{of}\;1\;\mathrm{if}\;\mathrm{the}\;\mathrm{participant}\;\mathrm{is}\;\mathrm{of}\;\mathrm{that}\;\mathrm{ethnicity}\;\mathrm{and}\;0\;\mathrm{if}\;\mathrm{not}\;\mathrm{of}\;\mathrm{that}\;\mathrm{ethnicity}\right).$$

Here, TG and GGT are the abbreviations of triglycerides and gamma-glutamyl transpeptidase, separately. In the calculation of USFLI, individuals are assigned to a value of 1 if they are classified as ‘non-Hispanic black’ or ‘Mexican American’ and 0 if they are not. Individuals with a FLI score ≥ 60 [31] or USFLI ≥30 [27] were defined as NAFLD.

#### The description of advanced liver fibrosis

Two groups of participants with NAFLD were categorized based on the NFS, FIB-4, and APRI scores. The formulas for these scores are as follows:

$$\mathrm{NFS}=-1.675+0.037\ast\;\mathrm{age}\;\left(\mathrm{years}\right)\;+\;0.094\;\ast\;\mathrm{BMI}\;\left(\mathrm{kg}/\mathrm m2\right)\;+\;1.13\;\ast\;\mathrm{IFG}/\mathrm{diab}e\mathrm{tes}\;\left(\mathrm{yes}=1,\;\mathrm{no}\;=\;0\right)\;+\;0.99\;\ast\;\mathrm{AST}/\mathrm{ALT}\;\mathrm{ration}\;-\;0.013\;\ast\;\mathrm{Platelet}\;\mathrm{counts}\;-\;0.66\;\ast\;\mathrm{albumin}\;\left(\mathrm g/\mathrm{dL}\right).$$

$$\mathrm{FIB}-4=\;\left(\mathrm{age}\;\ast\;\mathrm{AST}\right)/\left(\mathrm{Platelet}\;\mathrm{counts}\;\ast\;\left(\mathrm{SQRT}\left(\mathrm{ALT}\right)\right)\right)$$

$$\mathrm{APRI}=\left(\left[\mathrm{AST}/\mathrm{ULN}\right]/\mathrm{Platelet}\;\mathrm{counts}\right)\;\ast\;100.$$

AST and ALT stand for aspartate transaminase and alanine aminotransferase, respectively, while PLT represents platelet count. Participants’ NFS values are calculated as follows: 0 if they do not have diabetes or impaired glucose tolerance (IFG), and 1 if they do. The typical upper limit of AST is denoted by ULN in the APRI computation. In NAFLD patients, NFS > 0.676 or FIB-4 > 2.67 or APRI> 1.0 [28] were considered indicators of advanced liver fibrosis.

### Definition of lean/abdominal lean and obese/ abdominal obese individuals

**Fig. 2:**
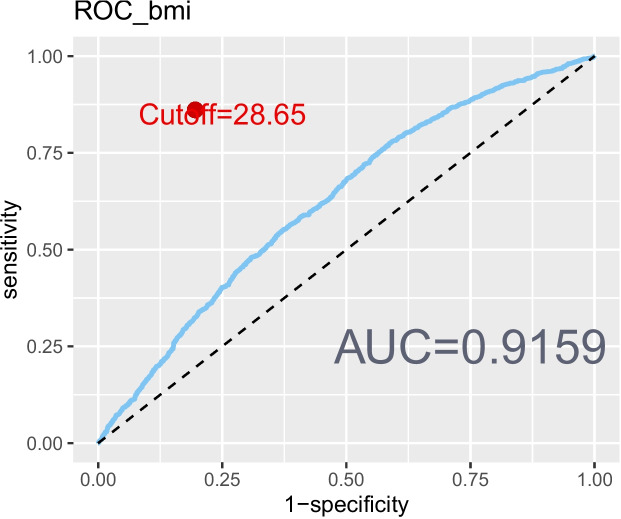
The ROC curve depicting the predictive ability of BMI for NAFLD

**Fig. 3:**
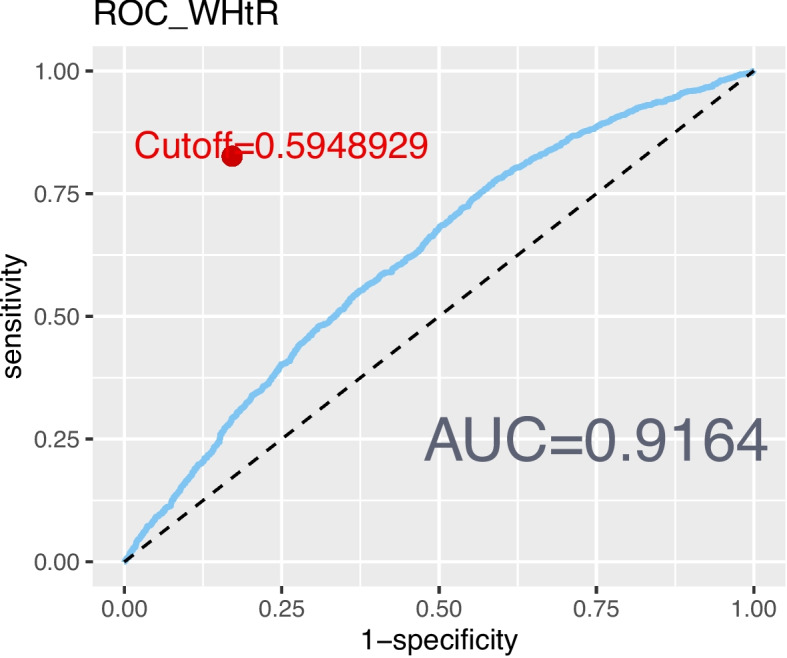
The ROC curve depicting the predictive ability of WHtR for NAFLD

**Fig. 4:**
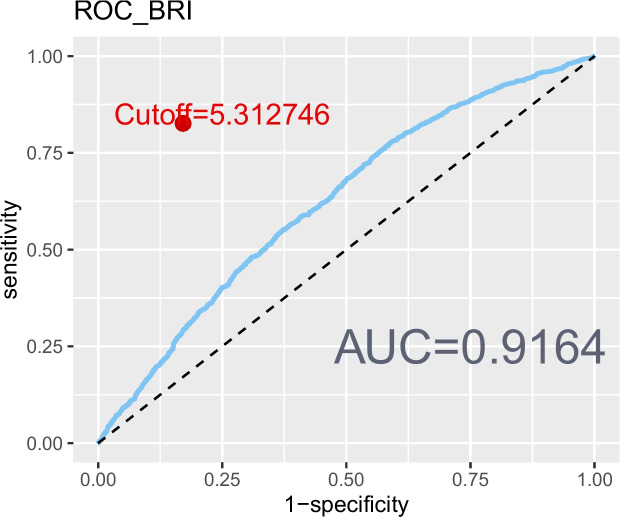
The ROC curve depicting the predictive ability of BRI for NAFLD

**Fig. 5:**
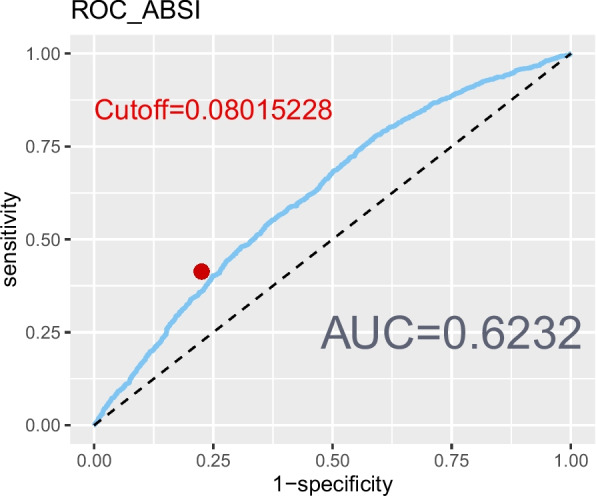
The ROC curve depicting the predictive ability of ABSI for NAFLD

The revised 2022 AGA Clinical Practice guidelines defined lean NAFLD as NAFLD occurring in individuals with a BMI < 25 kg/mˆ2 [32]. However, BMI alone may not provide a comprehensive assessment of body fat distribution [33]. Additional measures, such as the WHtR [8], body roundness index (BRI) [34], and a body shape index (ABSI) [35] were also incorporated to delineate abdominal obesity. To evaluate the correlation and reliability of these markers with NAFLD, ROC curves were created (Figs. 2, 3, 4, and 5). Among the measures considered, WHtR demonstrated both the best diagnostic performance and the simplest calculation method. Hence, a WHtR< 0.50 [36] was used as a measure of abdominal obesity, consistent with prior research. Finally, lean NAFLD was categorized as NAFLD with a BMI < 25.00 kg/mˆ2, while obese NAFLD was defined as having a BMI ≥ 25.00 kg/mˆ2. Additionally, abdominal-lean NAFLD was characterized by a WHtR< 0.5, whereas abdominal-obese NAFLD had a WHtR≥0.5.

### Dietary assessment

The creation and validation of DII has been documented elsewhere [18, 37]. DII was computed using 45 dietary factors collected from 11 different communities globally. A Z-score was calculated by normalizing each parameter’s value through the removal of the database mean and division by the parameter’s standard deviation. Then, by doubling and removing one (from − 1 to + 1 and centering on 0), the Z-score was transformed into percentile values [18, 38]. Each central percentile was then multiplied by its associated inflammatory impact score. Then, the DII scores for each dietary parameter were added to calculate an individual’s DII.

Dietary information in NHANSE was collected via a 24-hour recall interview done at the mobile examination center (MEC). In this research, a total of 28 different food parameters, including dietary calorie intake, protein, carbohydrates, cholesterol, fat, fatty acids (saturated, monounsaturated and polyunsaturated), folic acid, beta-carotene, ω-3 and ω-6 fatty acids, niacin, fiber, alcohol, caffeine, and various vitamins (A, B1, B2, B6, B12, C, D, E), iron, zinc, selenium, and magnesium were employed in the calculation of DII, consistent with prior studies [39–41]. After gathering the data, the DII for each parameter was computed using the following formula:

$$\mathrm D\mathrm I{\mathrm I}\_{\mathrm e}\mathrm a\mathrm c\mathrm h\;\mathrm{parameter}\;=\;\left[\left(\mathrm{Individual}'\mathrm s\;\mathrm i\mathrm n\mathrm t\mathrm a\mathrm k{\mathrm e}\_{\mathrm e}\mathrm a\mathrm c\mathrm h\;\mathrm{parameter}-\mathrm{Global}\;\mathrm{daily}\;\mathrm{mean}\;\mathrm i\mathrm n\mathrm t\mathrm a\mathrm k{\mathrm e}\_{\mathrm e}\mathrm a\mathrm c\mathrm h\;\mathrm{parameter}\right)/\mathrm{The}\;\mathrm{standard}\;\mathrm{deviation}\;\mathrm{of}\;\mathrm{global}\;\mathrm{daily}\;\mathrm m\mathrm e\mathrm a\mathrm n\;\mathrm{in}\mathrm t\mathrm a\mathrm k\mathrm e\;\mathrm{ea}\mathrm c\mathrm h\;\mathrm{parameter}\right]\ast\;\mathrm{The}\;\mathrm{inflammatory}\;\mathrm i\mathrm n\mathrm d\mathrm e\mathrm r{\mathrm x}_{\mathrm e}\mathrm a\mathrm c\mathrm h\;\mathrm{parameter}.-$$

After calculating the DII for each parameter, the DII scores of all parameters were summed to calculate an individual’s DII. Then, participants were divided into quartiles based on DII for further analyses. The quartiles were defined as follows: Quartile 1 (Q1): -5.20 < DII < − 0.09, Quartile 2 (Q2): -0.09 ≤ DII < 1.54, Quartile 3 (Q3): 1.54 ≤ DII < 2.90, and Quartile 4 (Q4): 2.90 ≤ DII < 5.52.

### Covariates

The main covariates in this study included demographic data such as age, gender, race, smoking habits, family income-to-poverty ratio (PIR), educational level, as well as laboratory examinations including cholesterol (TC), TG, albumin (ALB), ALT, AST, GGT, high-density lipoprotein (HDL), PLT, and other metabolic diseases such as hypertension and diabetes. Hypertension was defined as either: (a) a history of hypertension, or (b) systolic blood pressure (SBP) ≥ 140 mmhg, or (c) diastolic blood pressure (DBP) ≥ 90 mmhg. Diabetes was diagnosed if any of the following criteria were met: (a) a prior diagnosis of diabetes, (b) a hemoglobin A1C concentration (HbA1c) above 6.4%, or (c) a fasting plasma glucose level (FPG) over 125 mg/dL, or (d) the use of insulin.

### Statistical analysis

The statistical analysis in this study were conducted using R (4.3.1). Descriptive statistics were presented in various formats, including medians, averages, standard deviations, percentages and frequencies, depending on the data attributes. In Tables 1 and 2, the Chi-Square Test was utilized to examine the qualitative characteristics, while One-Way ANOVA and the Kruskal-Wallis H Test were performed to compare data from groups with normal or non-normal distributions, separately. Then, three logistic models were employed to calculate odds ratios (OR) and 95% confidence intervals (CI). Model 1 represented the original model with no confounding factors adjusted. Model 2 accounted for the impacts of age, gender, and races, while Model 3 further refined the education level, ratio of family income to poverty (PIR), hypertension, diabetes and smoking habits based on Model 2. Subgroup analyses based on age, gender, hypertension, and diabetes were conducted to assess the link between DII and NAFLD in diverse populations. All *P*-values were calculated on both sides and deemed statistically significant when below 0.05.

**Table 1:** Baseline characteristics of all participants according to quartiles of DII

	Q1	Q2	Q3	Q4	*P* value
	(*n* = 1289)	(*n* = 1282)	(*n* = 1293)	(*n* = 1288)	
Age (Years)	53.22 ± 16.40	52.82 ± 17.59	53.63 ± 17.12	53.31 ± 17.88	0.698
Gender (n, %)					< 0.001
Male	791 (61.40%)	687 (53.60%)	596 (46.10%)	489 (38.00%)	
Female	498 (38.60%)	595 (46.40%)	697 (53.90%)	799 (62.00%)	
Race (n, %)					< 0.001
Mexican American	162 (12.60%)	170 (13.30%)	151 (11.70%)	122 (9.50%)	
Non-Hispanic Asian	265 (20.60%)	183 (14.30%)	153 (11.80%)	94 (7.30%)	
Non-Hispanic Black	188 (14.60%)	219 (17.10%)	320 (24.70%)	348 (27.00%)	
Non-Hispanic White	498 (38.60%)	548 (42.70%)	502 (38.80%)	557 (43.20%)	
Other Hispanic	137 (10.60%)	124 (9.70%)	108 (8.40%)	115 (8.90%)	
Other Races	39 (3.00%)	38 (3.00%)	59 (4.60%)	52 (4.00%)	
Edu (n, %)					< 0.001
Below college	374 (29.00%)	458 (35.70%)	559 (43.20%)	703 (54.60%)	
College and above	915 (71.00%)	824 (64.30%)	734 (56.80%)	585 (45.40%)	
PIR	3.06 ± 1.67	2.75 ± 1.63	2.51 ± 1.55	2.12 ± 1.45	< 0.001
BMI (kg/mˆ2)	26.80 [23.80, 30.90]	28.30 [24.52, 32.30]	28.00 [24.50, 33.20]	29.40 [25.20, 34.80]	< 0.001
Waist (cm)	97.57 ± 15.41	100.07 ± 16.18	100.55 ± 16.85	102.94 ± 17.49	< 0.001
WHtR	0.57 [0.51, 0.63]	0.59 [0.53, 0.66]	0.59 [0.54, 0.67]	0.62 [0.55, 0.69]	< 0.001
SBP (mmhg)	123.96 ± 18.48	124.37 ± 17.48	126.24 ± 18.98	126.70 ± 20.59	< 0.001
DBP (mmhg)	70.51 ± 11.70	69.81 ± 12.62	70.39 ± 12.76	69.73 ± 13.66	0.294
TC (mg/dl)	187.63 ± 39.96	184.41 ± 40.89	189.04 ± 38.35	189.46 ± 45.40	0.008
TG (mg/dl)	117.30 ± 99.50	113.84 ± 77.68	117.27 ± 91.30	120.64 ± 101.21	0.328
HDL (mg/dl)	54.14 ± 15.94	52.83 ± 15.06	53.58 ± 15.31	51.45 ± 13.88	< 0.001
ALB (g/dl)	4.25 ± 0.31	4.19 ± 0.33	4.15 ± 0.34	4.10 ± 0.33	< 0.001
ALT (U/L)	24.07 ± 13.74	22.96 ± 12.28	23.07 ± 15.87	21.65 ± 16.13	< 0.001
AST (U/L)	24.44 ± 13.13	22.89 ± 8.33	23.66 ± 25.86	22.63 ± 25.07	0.084
GGT (U/L)	25.67 ± 22.93	25.15 ± 29.30	28.80 ± 33.92	29.18 ± 37.62	0.001
PLT (1000cells/μl)	226.48 ± 57.96	229.03 ± 59.39	231.98 ± 62.22	237.48 ± 65.10	< 0.001
Smoking_status					< 0.001
Never smoker	686 (65.00%)	657 (64.30%)	623 (60.40%)	561 (53.40%)	
Former smoker	298 (28.20%)	260 (25.40%)	251 (24.30%)	267 (25.40%)	
Current smoker	72 (6.80%)	105 (10.30%)	157 (15.20%)	222 (21.10%)	
Hypertension (n, %)					< 0.001
Yes	583 (45.20%)	567 (44.20%)	620 (48.00%)	679 (52.70%)	
No	706 (54.80%)	715 (55.80%)	673 (52.00%)	609 (47.30%)	
Diabetes (n, %)					< 0.001
Yes	251 (19.50%)	279 (21.80%)	330 (25.50%)	350 (27.20%)	
No	1038 (80.50%)	1003 (78.20%)	963 (74.50%)	938 (72.80%)	
NAFLD (n, %)					< 0.001
Yes	532 (41.30%)	617 (48.10%)	635 (49.10%)	719 (55.80%)	
No	757 (58.70%)	665 (51.90%)	658 (50.90%)	569 (44.20%)	
Advanced_fibrosis (n, %)					0.002
Yes	109 (20.60%)	166 (27.00%)	164 (25.80%)	217 (30.20%)	
No	421 (79.40%)	448 (73.00%)	471 (74.20%)	502 (69.80%)	
DII	−1.31 [−2.21, −0.65]	0.78 [0.36, 1.18]	2.20 [1.89, 2.54]	3.62 [3.26, 4.02]	< 0.001

*Abbreviations: DII* dietary inflammatory index. Q1: -5.20 < DII < −0.09. Q2: -0.09 ≤ DII < 1.54. Q3: 1.54 ≤ DII < 2.90. Q4: 2.90 ≤ DII < 5.52, *NAFLD* non-alcoholic fatty liver disease, *Advanced_fibrosis *patients were grouped according to whether they were defined as advanced liver fibrosis or not, *PIR *ratio of family income to poverty, *Edu* educational level, *BMI* body mass index(kg/mˆ2), *Waist *waist circumference(cm), *Height *standing height(cm), *WHtR *ratio of waist circumference to standing height, *SBP *systolic blood pressure(mmhg), *DBP* diastolic blood pressure(mmhg), *TC* total cholesterol(mg/dL), *TG* triglyceride(mg/dL), *HDL* high-density lipoprotein cholesterol(mg/dL), *ALB* Albumin(g/dL), *ALT* alanine aminotransferase(U/L), *AST* aspartate transaminase(U/L), *GGT* gamma-glutamyl transferase(U/L), *PLT* platelet count(1000cells/μl), *Smoking_status *participants were grouped by smoking status acquired by questionnaire

**Table 2:** Characteristics of lean NAFLD and obese NAFLD

	Lean NAFLD	Obese NAFLD	*P* value
N, %	86	2417	–
Age (Years)	62.72 ± 12.26	55.26 ± 16.05	< 0.001
Gender (n, %)			0.843
Male	46 (53.50%)	1252 (51.80%)	
Female	40 (46.50%)	1165 (48.20%)	
Race (n, %)			< 0.001
Mexican American	12 (14.00%)	368 (15.20%)	
Non-Hispanic Asian	30 (34.90%)	172 (7.10%)	
Non-Hispanic Black	6 (7.00%)	493 (20.40%)	
Non-Hispanic White	29 (33.70%)	1055 (43.60)	
Other Hispanic	8 (9.30%)	231 (9.60%)	
Other Races	1 (1.20%)	98 (4.10%)	
Edu (n, %)			0.991
Below college	39 (45.30%)	1080 (44.70%)	
College and above	47 (54.70%)	1337 (55.30%)	
PIR	2.73 ± 1.56	2.46 ± 1.58	0.110
BMI (kg/mˆ2)	23.90 [23.22,24.58]	32.90 [29.90,37.40]	< 0.001
WHtR	0.54 [0.53,0.58]	0.67 [0.62,0.72]	< 0.001
SBP (mmhg)	129.67 ± 16.90	128.32 ± 17.81	0.488
DBP (mmhg)	65.74 ± 18.42	71.71 ± 13.19	< 0.001
TC (mg/dl)	193.73 ± 45.32	189.21 ± 42.10	0.329
TG (mg/dl)	151.94 ± 73.17	149.92 ± 117.21	0.874
HDL (mg/dl)	51.43 ± 12.86	47.07 ± 11.78	0.001
ALB (g/dl)	4.20 ± 0.38	4.11 ± 0.33	0.007
ALT (U/L)	29.20 ± 20.83	26.42 ± 17.20	0.144
AST (U/L)	26.29 ± 11.78	24.57 ± 26.14	0.548
GGT (U/L)	67.09 ± 70.58	34.39 ± 39.05	< 0.001
Smoking_status			0.202
Never smoker	36 (47.40%)	1098 (56.80%)	
Former smoker	26 (34.20%)	585 (30.30%)	
Current smoker	14 (18.40%)	250 (12.90%)	
Hypertension (n, %)			0.828
Yes	50 (58.10%)	1448 (59.90%)	
No	36 (41.90%)	969 (40.10%)	
Diabetes (n, %)			0.031
Yes	42 (48.80%)	890 (36.80%)	
No	44 (51.20%)	1527 (63.20%)	
Advanced liver fibrosis (n, %)			0.001
Yes	8 (9.50%)	648 (26.80%)	
No	76 (90.50%)	1766 (73.20%)	
DII	1.75 [−0.43,2.98]	1.74 [0.23,3.12]	0.471
Quartiles of DII (n, %)			0.253
Q1	24 (27.90%)	508 (21.00%)	
Q2	15 (17.40%)	602 (24.90%)	
Q3	24 (27.90%)	611 (25.30%)	
Q4	23 (26.70%)	696 (28.80%)	

Abbreviations: *NAFLD* non-alcoholic fatty liver disease, *Lean NAFLD *Individuals diagnosed with NAFLD and with a BMI ≤ 25.00 kg/mˆ2, *Obese NAFLD *Individuals diagnosed with NAFLD and with a BMI > 25.00 kg/mˆ2, *Advanced_fibrosis *patients were grouped according to whether they were defined as advanced liver fibrosis or not, *BMI* body mass index. *Edu* educational level, *PIR *ratio of family income to poverty, *WHtR *ratio of waist circumference to standing height, *SBP* systolic blood pressure, *DBP* diastolic blood pressure, *TC* total cholesterol, *TG* triglyceride, *HDL* high-density lipoprotein cholesterol, *ALB* Albumin, *ALT* alanine aminotransferase, *AST* aspartate transaminase, *GGT* gamma-glutamyl transferase, *PLT* platelet count(1000cells/μl). Smoking_status: participants were grouped by smoking status acquired by questionnaire

## Results

### The features of participants

Table 1 presents the baseline characteristics of the 5152 individuals grouped by DII quartiles. Among them, 1289 individuals were assigned to Group Q1, 1282 to Group Q2, 1293 to Group Q3 and 1288 to Group Q4. Significant variations were observed among the DII groups concerning gender, race, education level, PIR, smoking habits, BMI, waist circumstance, WHtR, SBP, TC, HDL, ALB, ALT, GGT, PLT, hypertension, diabetes, NAFLD as well as advanced liver fibrosis (*P* < 0.05). These disparities more pronounced with increasing DII scores.

Table 2 provides a comprehensive description of both lean and obese NAFLD. Among the 2503 NAFLD patients, 86 were classified as lean NAFLD while 2417 as obese NAFLD. Lean NAFLD patients tended to be older, with lower DBP, and higher levels of HDL and GGT. Additionally, they exhibited a higher percentage of diabetes (lean NAFLD vs obese NAFLD = 48.80% vs 36.80%), and a lower percentage of advanced liver fibrosis (lean NAFLD vs obese NAFLD = 9.50% vs 26.80%). Moreover, notable racial differences could be observed between lean and obese NAFLD. Non-Hispanic Asians exhibited a higher risk of being lean NAFLD, whereas Non-Hispanic Whites were more likely to suffer from obese NAFLD.

### DII levels and NAFLD

Table 3 displays the detailed information on the association between DII and NAFLD in multivariable logistic regression models, as previously discussed. In general, NAFLD correlates positively with higher DII in all three models. The ORs of Q4 are 1.80 [1.54,2.10], 1.86 [1.57,2.19], 1.81 [1.48, 2.21], in the Model 1, Model 2, Model 3, separately. All *P*-values were below *0.05*.

**Table 3:** Association between quartiles of DII and NAFLD/advanced liver fibrosis

	Q1		Q2		Q3		Q4	
	OR (95%CI)	*P* value	OR (95%CI)	*P* value	OR (95%CI)	*P* value	OR (95%CI)	*P* value
**NAFLD**								
Model 1	Reference		1.32 (1.13,1.54)	< 0.001	1.37 (1.18,1.60)	< 0.001	1.80 (1.54,2.10)	< 0.001
Model 2	Reference		1.31 (1.12,1.54)	< 0.001	1.39 (1.18,1.63)	< 0.001	1.86 (1.57,2.19)	< 0.001
Model 3	Reference		1.27 (1.05,1.53)	0.015	1.26(1.04,1.53)	0.017	1.81 (1.48,2.21)	< 0.001
**Advanced fibrosis**								
Model 1	Reference		1.43 (1.09,1.89)	0.011	1.34 (1.02,1.77)	0.035	1.67 (1.28,2.17)	< 0.001
Model 2	Reference		1.46 (1.07,1.99)	0.016	1.30 (0.96,1.77)	0.092	1.73 (1,28,2.34)	< 0.001
Model 3	Reference		1.45 (1.01,2.06)	0.042	1.28 (0.90,1.83)	0.169	1.46 (1.02,2.07)	0.037

Model 1: no covariates were adjusted

Model 2: Age, gender and race were adjusted

Model 3: Age, gender, race, education level, PIR, smoking status, hypertension, diabetes was adjusted

Furthermore, the correlation between DII and advanced liver fibrosis was assessed using the same methodology. Overall, a notable positive relationship was observed between higher DII and advanced liver fibrosis in all three models, especially in the highest DII group (Model 1: OR = 1.67 [1.28,2.17]; Model 2: OR = 1.73 [1.28, 2.34]; Model 3: OR = 1.46 [1.02,2.07]).

### Subgroup analysis

Table 4 summarizes the findings of the subgroup analysis. Higher DII was related with an increased likelihood of NAFLD in adults both below and above the age of 60 (Q4: age ≤ 60, OR = 1.80 [1.39,2.33];age > 60, OR = 1.88 [1.37, 2.60]). Generally, a higher DII elevated the risk of NAFLD in both genders, particularly in females (in group Q4: male: OR = 1.35 [1.02,1.80], female: 2.35 [1.74,3.17]). Therefore, the findings imply that women may be more vulnerable to dietary inflammation than men. Surprisingly, among those without hypertension or diabetes, DII was more favorably related to the risk of NAFLD.

**Table 4:** Subgroup analyses between quartiles of DII and NAFLD

	Quartiles of DII							
	Q1		Q2		Q3		Q4	
	OR (95%CI)	*P* value	OR (95%CI)	*P* value	OR (95%CI)	*P* value	OR (95%CI)	*P* value
**Age**^**1**^								
≤60 (*n* = 3139)	Reference		1.43 (1.12,1.82)	0.005	1.16 (0.90,1.49)	0.256	1.80 (1.39,2.33)	< 0.001
> 60 (*n* = 2013)	Reference		1.06 (0.78,1.44)	0.712	1.46 (1.07,1.99)	0.018	1.88 (1.37,2.60)	< 0.001
**Gender**^**2**^								
Male (*n* = 2563)	Reference		1.13 (0.88,1.44)	0.338	1.37 (1.05,1.78)	0.019	1.35 (1.02,1.80)	0.036
Female (*n* = 2589)	Reference		1.58 (1.16,2.14)	0.004	1.32 (0.98,1.78)	0.070	2.35 (1.74,3.17)	< 0.001
**Diabetesª**								
Yes (*n* = 1210)	Reference		0.81 (0.51,1.31)	0.391	1.01 (0.63,1.61)	0.976	1.46 (0.89,2.39)	0.133
No (*n* = 3942)	Reference		1.40 (1.13,1.73)	0.002	1.31 (1.06,1.63)	0.014	1.90 (1.52,2.37)	< 0.001
**Hypertension°**								
Yes (*n* = 2449)	Reference		1.30 (0.97,1.72)	0.076	1.03 (0.78,1.37)	0.829	1.59 (1.19,2.13)	0.002
No (*n* = 2703)	Reference		1.74 (1.07,2.82)	0.025	1.57 (0.92,2.66)	0.095	2.11 (1.20,3.70)	0.009

^1^Adjusted by gender, race, education level, PIR, smoking status, hypertension, diabetes

^2^Adjusted by age, race, education level, PIR, smoking status, hypertension, diabetes

^a^Adjusted by age, gender, race, education level, pir, smoking status, hypertension

**°**Adjusted by age, gender, race, education level, pir, smoking status, diabetes

### The link between DII and lean/abdominal lean NAFLD

Table 5 contains comprehensive information regarding the relationship between DII and lean or abdominal lean NAFLD across three logistic regression models. Model 1 remained unaltered, while Model 2 was modified for age and gender. Model 3 incorporated corrections for educational level, PIR, smoking habits, hypertension and diabetes based on the adjustments made in Model 2. Obviously, no statistical association could be observed between DII and lean or abdominal lean NAFLDs. However, a favorable correlation was identified between DII and obese or abdominal obese NAFLD (For BMI ≥25.00 kg/mˆ2, Q4: model 3: OR = 1.56 [1.23,1.98]. For WHtR≥0.5, Q4: model 3: OR = 1.48 [1.23,1.79]).

**Table 5:** Association between quartiles of DII and lean/obese NAFLD (divided by BMI and WHtR)

	Model 1		Model 2		Model3	
	OR (95%CI)	*P* value	OR (95%CI)	*P* value	OR (95%CI)	*P* value
BMI < 25.00(kg/m^2)						
Q1 (*n* = 450)	Reference		Reference		Reference	
Q2 (*n* = 359)	0.77 (0.40,1.50)	0.447	0.78 (0.40,1.53)	0.471	0.56 (0.26,1.22)	0.143
Q3 (*n* = 364)	1.25 (0.70,2.25)	0.449	1.26 (0.69,2.28)	0.451	0.99 (0.50,1.97)	0.972
Q4 (*n* = 310)	1.42 (0.79,2.57)	0.243	1.51 (0.82,2.79)	0.182	1.25 (0.61,2.57)	0.537
BMI ≥ 25.00(kg/m^2)						
Q1 (*n* = 839)	Reference		Reference		Reference	
Q2 (*n* = 923)	1.22 (1.01,1.48)	0.043	1.26 (1.03,1.53)	0.022	1.19 (0.95,1.50)	0.129
Q3 (*n* = 929)	1.25 (1.03,1.52)	0.023	1.31 (1.08,1.59)	0.007	1.19 (0.94,1.49)	0.147
Q4 (*n* = 978)	1.61 (1.32,1.96)	< 0.001	1.73 (1.42,2.11)	< 0.001	1.56 (1.23,1.98)	< 0.001
WHtR≤0.50						
Q1 (*n* = 260)	Reference		Reference		Reference	
Q2 (*n* = 197)	0.00 (0.00, Inf)	0.990	0.00 (0.00, Inf)	0.993	0.00 (0.00, Inf)	0.992
Q3 (*n* = 179)	1.46 (0.29,7.32)	0.645	1.23 (0.24,6.44)	0.803	0.77 (0.12,4.84)	0.778
Q4 (*n* = 143)	1.22 (0.20,7.36)	0.832	1.57 (0.25,9.85)	0.630	0.52 (0.06,4.65)	0.556
WHtR> 0.50						
Q1 (*n* = 1029)	Reference		Reference		Reference	
Q2 (*n* = 1085)	1.25 (1.05,1.48)	0.012	1.28 (1.08,1.52)	0.005	1.26 (1.05,1.51)	0.012
Q3 (*n* = 1114)	1.24 (1.05,1.47)	0.014	1.31 (1.10,1.56)	0.002	1.19 (0.99,1.43)	0.059
Q4 (*n* = 1145)	1.58 (1.33,1.88)	< 0.001	1.73 (1.45,2.06)	< 0.001	1.48 (1.23,1.79)	< 0.001

Model 1: no covariates were adjusted

Model 2: Age and gender were adjusted

Model 3: Age, gender, education level, PIR, smoking status, hypertension, diabetes were adjusted

## Discussion

Plenty of studies have demonstrated the adverse effects of pro-inflammatory diets on various metabolic diseases, including hypertension [42], heart failure [43], cognitive impairment [39] and diabetes [41]. More importantly, dietary inflammation has been implicated in the development of fatty liver disease [22, 44]. However, further investigation into the relationship of DII and NAFLD is warranted. On the one hand, it’s crucial to exclude excessive alcohol consumption as both a significant contributor to dietary inflammation and another leading cause of fatty liver disease. Neither Ting Tian [22] nor Mohsen Mazidi [44] definitively ruled out the direct effect of excessive alcohol intake in their studies due to their emphasis on a spectrum of fatty liver conditions. On the other hand, recent research has identified two subtypes of lean NAFLD. Type 1 is more common in those with abdominal obesity and insulin resistance (IR), while type 2 is more commonly observed in those with monogenic disorders [45, 46]. While adopting healthy eating habits is recommended for all forms of NAFLD [47], clinicians face challenges in selecting appropriate clinical assessments for patients with varied weights and body types. Yet, limited research has explored the effect of diet on NAFLD in individuals with varying body shapes. This knowledge gap inspired this study.

The main conclusions of this investigation are as follows: Firstly, individuals adhering to a pro-inflammatory diet are more susceptible to NAFLD and advanced liver fibrosis. Secondly, higher DII scores correlate with elevated BMI and WHtR. Moreover, the impact of dietary inflammation appears less pronounced in lean NAFLD compared to obese NAFLD. Finally, subgroup analysis indicates that female participants, and those without diabetes are particularly vulnerable to developing NAFLD when consuming a pro-inflammatory diet. The findings are consistent with earlier research revealing the induction of chronic inflammation by diet [13, 22, 48] and its role in the development of NAFLD [49].

In general, diets with higher DII are associated with processed foods containing increased calories, fat, cholesterol, and carbohydrates. More importantly, poor dietary habits often coincide with the accumulation of subcutaneous and visceral fat [40]. Initially, a pro-inflammatory diet stimulates adipose tissue to produce pro-inflammatory adipokines and cytokines, including TNF-α, IL-1, IL-6, etc. [50, 51]. These compounds contribute to persistent low-grade inflammation, a common etiology in both obesity and NAFLD. Subsequently, these pro-inflammatory mediators increase the production of reactive oxygen and nitrogen species [52], and induce immunological dysfunction by altering macrophages [53, 54], thereby exacerbating liver damage. Moreover, high DII diets have been linked to insulin resistance (IR) [41, 55] and the modification of gut flora [56]. Additionally, liver tissue exposure to prolonged free fatty acids (FFA) [57], one of the primary causes of NAFLD [58], is more common in obese individuals. Thus, it is hypothesized that obesity, particularly abdominal obesity, mediates the development of NAFLD driven by pro-inflammatory diets. Conversely, dietary factors have a lesser impact on lean NAFLD, highlighting the importance of genetic and epigenetic factors in the onset and progression of lean NAFLD. Previous studies have supported this assumption, demonstrating that certain genetic variations, such as the G variation in PNPLA3 and the T variant in TM6SF2, are more prevalent among lean NAFLD patients [59], potentially impacting processes related to inflammation, oxidative stress and lipid metabolism [60–62]. In a word, obese individuals may benefit more from modifying their dietary habits to prevent NAFLD, whereas lean individuals may require more targeted pharmacological therapies focusing on genes and downstream pathways rather than relying solely on dietary interventions. These therapies may include the use of certain anti-sense oligonucleotides, RNA interference, and medicines regulating gut flora [63]. Encouragingly, various drugs aimed at these processes are currently undergoing clinical trials, including traditional Chinese therapies such as Huazhi Fugan Granules [64], Fufang Zhenzhu Tiaozhi formula (FTZ) [65] and Chaihu-Shugan-San, Shen-Ling-Bai-Zhu-San [66].

In subgroup analyses, women were observed to have higher DII diets and be more vulnerable to the adverse effects of DII. This gender-related difference may be attributed to variations in dietary patterns and food choices. Besides, women are more likely to weight gain, especially around menopause, when estrogen levels decline, leading to increased fat storage [67]. Furthermore, research suggests that females are less likely to be physically active and are more prone to overeat owing to lifestyle and emotional factors [68, 69], highlighting the necessity of optimizing diet structure in women. Surprisingly, in this study, DII exhibits a stronger favorable correlation with NAFLD among participants without hypertension or diabetes. This phenomenon persists even after re-testing. One possible explanation is that those with hypertension and diabetes are already metabolically impaired, displaying decreased insulin sensitivity. Consequently, modifying the existing metabolic dysfunction remains challenging even with dietary improvements. Moreover, hypertension and diabetes serve as both causes and significant consequences of NAFLD, potentially leading to collinearity issues in data processing. This may also be due to predisposition and other lifestyle variables, such as varying levels of activity or quality of sleep and so on. To sum up, even individuals who have not been diagnosed with hypertension or diabetes should adopt a healthy diet pattern. This not only reduces the chance of acquiring hypertension and diabetes, but also mitigates the possibility of NAFLD.

### Strengths and limitations

A major highlight of this study is its wide scope and thorough preparation, conducted within an organized multistage and cross-sectional project supervised by the NCHS. Furthermore, the program’s broad inclusiveness, which includes racial diversity, ensures a robust and representative sample, thereby enhancing the dependability and quality of this research. However, some restrictions should be acknowledged. Firstly, diagnostic uncertainty might arise owing to lack of a clear strategy or imaging data in detecting NAFLD and advanced liver fibrosis. Secondly, the use of questionnaires to collect data on dietary components may introduce recollection bias. Finally, despite the best efforts to adjusting for confounding variables, the potential influence of certain macronutrient-related confounders cannot be entirely avoided. However, the conclusions drawn in this study are considered valid and hold significant importance due to the researchers’ attempts to mitigate the impact of extraneous variables.

## Conclusion

To sum up, this study demonstrated a notable positive connection between DII and NAFLD, as well as its’ progression to advanced liver fibrosis. Significantly, the impact of dietary inflammation on NAFLD was more pronounced in obese individuals compared to their lean counterparts. Furthermore, female participants, and those without a diagnose of hypertension and diabetes were more vulnerable to the negative effects of pro-inflammatory diets. The clinical significance or this study is multifaced: Firstly, it highlights the importance of dietary management in the general population, recommending diets with lower DII. Secondly, the study suggests that obese individuals may benefit more from adopting healthier eating patterns. Conversely, lean individuals may require more targeted pharmacological therapies on genes and their downstream pathways, indicating the need for further research in this area. Finally, the findings emphasize the importance of tailored dietary recommendations for specific demographic groups, such as female and those without hypertension or diabetes, to promote public health and prevent NAFLD.

## Supplementary Information

**Supplementary Material 1.**

**Supplementary Material 2.**

## Data Availability

No datasets were generated or analysed during the current study.
