# Supplementary figures and images for: Dietary inflammatory impact on NAFLD development in obese vs. lean individuals: an analysis based on NHANES 2003–2018
Source: Lipids Health Dis. 2024 Apr 29;23:127. doi: 10.1186/s12944-024-02082-4 (PMC11619212; doi:10.1186/s12944-024-02082-4)

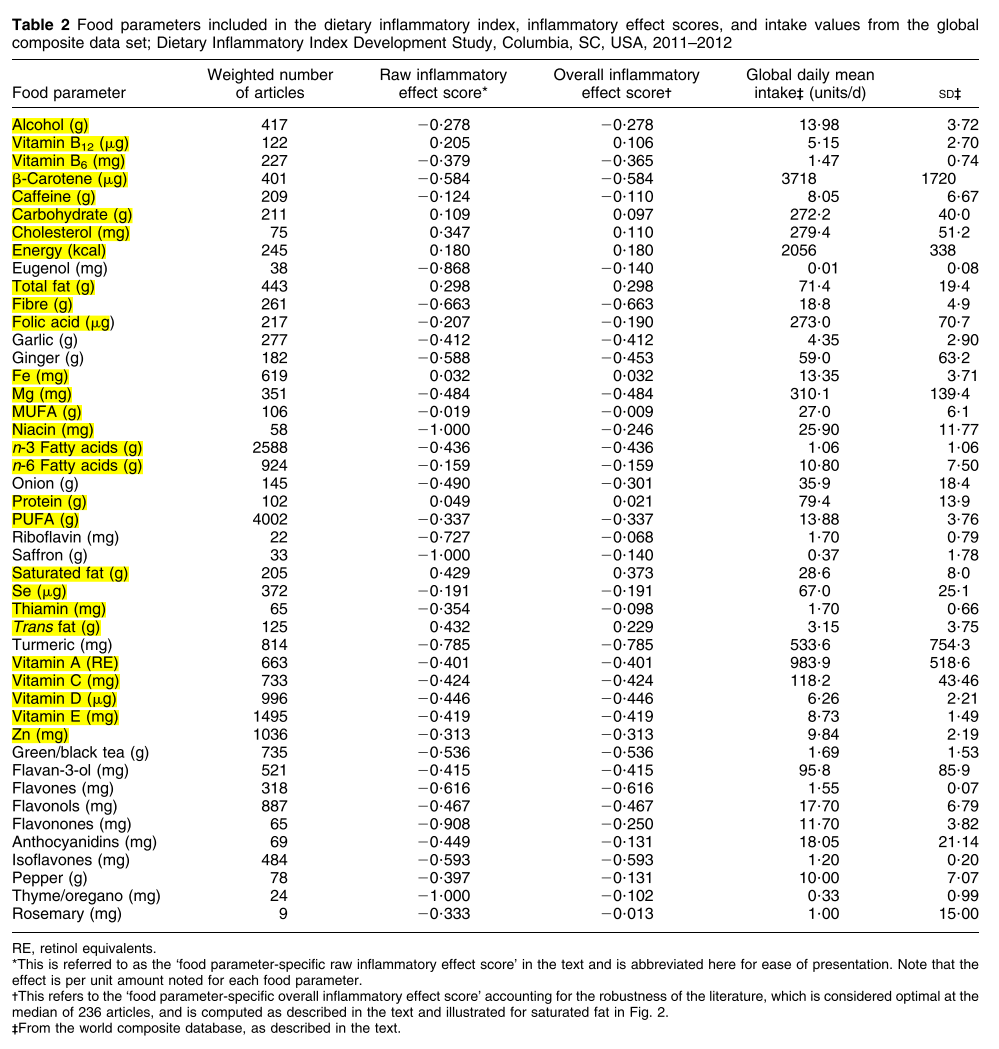

Supplement: Supplementary file 1 — Supplementary Material 1. [file 12944_2024_2082_MOESM1_ESM.docx]
