# Supplementary material for: Dietary inflammatory impact on NAFLD development in obese vs. lean individuals: an analysis based on NHANES 2003–2018
Source: Lipids Health Dis. 2024 Apr 29;23:127. doi: 10.1186/s12944-024-02082-4 (PMC11619212; doi:10.1186/s12944-024-02082-4)
Supplement: Supplementary file 2 — Supplementary Material 2. [file 12944_2024_2082_MOESM2_ESM.pdf]

20240221180036957740673330475008

WORD COUNT 5235

TIME SUBMITTED 21-FEB-2024 06:01PM

PAPER ID 107048641

Dietary Inflammatory Impact on NAFLD Development in Obese vs. Lean Individuals: an analysis based on NHANES 2003-2018 Analysis.

Abstract

Background

Non-alcoholic fatty liver disease (NAFLD), which is commonly linked with obesity, can also affect individuals of normal weight, a condition known as "lean NAFLD" and impose similar burdens and negative effects. However, the impact of diet on lean NAFLD remains underexplored. Our objective is to find out how the Dietary Inflammatory Index (DII) and NAFLD correlate, in the Americans, stratified by waist-to-height ratio (WHtR) together with body mass index (BMI).

Methods

5,152 participants from the National Health and Nutrition Examination Survey (NHANES) 2003-2018 were comprised in the final analysis. Serological markers were used to define both NAFLD and advanced liver fibrosis. Lean and abdominal lean individuals were identified using BMI and WHtR, separately. DII was determined by assigning scores to 28 distinct food parameters based on their inflammatory potential, obtained from the NAHNES website. Differences across DII quartiles were evaluated using the Kruskal-Wallis H Test, Chi-Square Test along with One-Way ANOVA. The correlation between DII and NAFLD was determined by multiple regression models and subgroup analyses.

Results

Among the 5,152 subjects, 2,503 were diagnosed with NAFLD, with 86 cases of lean NAFLD and 8 cases of abdominal lean NAFLD. DII was shown to be positively linked with NAFLD (Odds Ratio (OR)=1.81 [1.48-2.21], *P < 0.001)* and advanced liver fibrosis (OR=1.46 [1.02-2.07], *P = 0.037*). Further analysis revealed that this association was primarily observed in obese/abdominal obese participants (In BMI ⩾ 25.00 kg/m^2, OR=1.56 [1.23-1.98], *P <0.001*. In WHtR>0.50, OR=1.48 [1.23-1.79], *P <0.001*.) rather than their lean counterparts. Subgroup analyses demonstrated that female individuals without a diagnosis of hypertension or diabetes appeared to be more sensitive to the rise in DII.

Conclusions

Our data demonstrated a substantial positive correlation between DII and NAFLD in general population. The impact of a pro-inflammatory diet appeared to be less prominent in lean individuals compared to obese ones.

Keywords:

DII, NAFLD, lean individuals, abdominal obesity, BMI, WHtR.

Introduction

Non-alcoholic fatty liver disease (NAFLD) is described as the deposit of extra fat in the liver without significant alcohol consumption or other long-term liver illnesses, including viral hepatitis or genetic liver disorders [1, 2]. Nowadays, NAFLD is the

major cause of end-stage liver disease, primary liver carcinoma and demand for liver replacement surgery, placing a significant burden worldwide [3]. In addition to its well-established association with obesity, 'lean NAFLD' refers to NAFLD that affects individuals of normal weight [4, 5]. The incidence of lean NAFLD varies by region and races. A meta-analysis showed that lean NAFLD approximately accounted for 19.20% of NAFLD patients worldwide (95% CI:3.70-7.00) [6]. Previous studies have indicated that lean NAFLD may exhibit comparable outcomes to conventional NAFLD or potentially even worse liver-related events and overall mortality [7]. To date, genetics, epigenetics, dietary factors, and physical exercise have all been linked to the onset of NAFLD in lean individuals by influencing metabolic flexibility and adaptability [8, 9]. However, the specific mechanisms of lean NAFLD remain unclear. Additionally, specific guidelines for lean NAFLD are absent.

Recent study has demonstrated the critical function of inflammation in the onset of NAFLD [10, 11]. In humans, the inflammatory balance is maintained by cytokines including IL-6 and IL-1, along with tumor necrosis factor-alpha (TNF-α) and C-reactive protein (CRP) [12]. Disruption of this balance may lead to mild persistent inflammation and tissue damage. Diet is a key factor in modifying the inflammatory state in humans and has been widely used in NAFLD treatment. It is recommended to minimize the consumption of a typical Western eating style while promoting the adoption of a Mediterranean diet, which includes a higher intake of omega-3 as well as monounsaturated fatty acids and a lower intake of carbohydrates, refined carbs, and sweets [13-17]. To offer the public with more precise dietary guidance, Shivappa et al

devised the Dietary Inflammatory Index (DII), which is currently widely utilized to quantify the impact of an individual's diet on inflammation [18]. Previous research has revealed the substantial link between DII and obesity [19], type 2 diabetes (T2DM) [20], hypertension [21] and metabolic dysfunction-associated fatty liver disease (MAFLD) [22]. However, research on the relationship between DII and NAFLD in individuals with varying weights and body shapes is limited.

Nowadays, several non-invasive serologic tests are widely used as in diagnosing NAFLD and advanced liver fibrosis. These tests include the Fatty Liver Index (FLI), the US Fatty Liver Index (USFLI), the Non-Alcoholic Fatty Liver Disease Fibrosis Score (NFS), the Fibrosis 4 Index (FIB-4), the Hepatic Steatosis Index (HSI) and the Aspartate Aminotransferase /Platelet Ratio Index (APRI). Numerous epidemiological studies [23-29] have confirmed the validity of these markers. Our objective is to explore associations between DII and NAFLD in different body mass index (BMI) and body shapes (defined by waist-to-height ratio (WHtR)), aiming to provide more detailed dietary advice for NAFLD.

Methods

Population and study design

NHANES, a comprehensive database which tracks the nutritional status and health of the Americans [30], is administered by the Centers for Disease Control and Prevention (CDC). The data for this study were obtained from the eight NHANES cycles spanned from 2003 to 2018, as they included all of the relevant variables,

which were all available on the NHANES official website. The initial dataset consisted of 80,312 individuals. Participants were excluded if they were: (1) under the age of 18, (2) pregnant or were unable to submit a urine sample for testing, (3) had other chronic liver diseases (hepatitis B, C and liver carcinoma), (4) had excessive alcohol consumption, (5) had incomplete information on crucial factors, including dietary data, demographic, laboratory and questionnaire. Following this screening procedure, the final research comprised 5,152 individuals (Figure 1 is our study design).

Diagnostic Criteria and definition

Definition of NAFLD

As previously stated, FLI and USFLI jointly defined NAFLD. The formula is shown below:

$$FLI = \left(\frac{e^{0.953\times\log_e(TG)+0.139\times BMI+0.718\times\log_e(GGT)+0.053\times waist\ \text{circumference}-15.745}}{1+e^{0.953\times\log_e(TG)+0.139\times BMI+0.718\times\log_e(GGT)+0.053\times waist\ \text{circumference}-15.745}}\right)\bullet 100$$

$$USFLI = \left(\frac{e^{0.3458\times Mexican\ \text{American}-0.8073\times\text{non-Hispanic black}+0.0093\times\text{age}+0.6151\times\log_e(GGT)+0.0249\times waist\ \text{circumference}+1.1792\times\log_e(insulin)+0.8242\times\log_e(glucose)-14.7812}}{1+e^{0.3458\times Mexican\ \text{American}-0.8073\times\text{non-Hispanic black}+0.0093\times\text{age}+0.6151\times\log_e(GGT)+0.0249\times waist\ \text{circumference}+1.1792\times\log_e(insulin)+0.8242\times\log_e(glucose)-14.7812}}\right)\times 100$$

Here, TG and GGT are the abbreviations of triglycerides and gamma-glutamyl transpeptidase, separately. In the calculation of USFLI, individuals are assigned to a value of 1 if they are classified as 'non-Hispanic black' or 'Mexican American' and 0 if they are not. Individuals with a FLI score ≥60 [31] or USFLI ≥30 [27] were defined as NAFLD.

The description of advanced liver fibrosis

Two groups of participants with NAFLD were created based on the NFS, FIB-4,

and APRI scores. The following are the formulas:

$$NFS = -1.675 + 0.037 \times age + 0.094 \times BMI + 1.13 \times IFG \text{ or } diabetes(yes = 1, no = 0) + 0.99 \times AST / ALT - 0.013 \times PLT - 0.66 \times Alb$$

$$FIB-4 = \frac{Age \times AST}{PLT \times \sqrt{ALT}}$$

$$APRI = (\frac{AST / ULN}{PLT}) \times 100$$

AST and ALT are initials for aspartate transaminase and alanine aminotransferase, respectively. While PLT is an acronym for platelet count. Participants' NFS values are calculated as follows: 0 if they do not have diabetes or impaired glucose tolerance (IFG), and 1 if they do. The typical upper limit of AST is denoted by ULN in the APRI computation. In NAFLD patients, NFS>0.676 or FIB-4>2.67 or APRI>1.0 [28] were deemed indicators of advanced liver fibrosis,

Definition of lean/abdominal lean and obese/ abdominal obese individuals

The revised 2022 AGA Clinical Practice guidelines defined lean NAFLD as NAFLD in individuals with a BMI <25 kg/m^2 [32]. However, BMI alone may not provide a comprehensive assessment of body fat distribution [33]. Additional measures , such as the WHtR [8], body roundness index (BRI) [34], and a body shape index (ABSI) [35] were also included to delineate abdominal obesity. To evaluate the correlation and reliability of these markers with NAFLD, ROC curves were created (Figure 2-Figure5). WHtR and BRI appeared to be more accurate in predicting NAFLD. Hence, WHtR<0.50 [36] was used as a measurement of abdominal obesity, consistent with prior research. Finally, lean NAFLD was categorized as NAFLD with a BMI<25.00 kg/m^2, while obese NAFLD as having a BMI⩾25.00 kg/m^2. Meanwhile, abdominal-lean NAFLD was characterized as NAFLD having a

WHtR<0.5, whereas abdominal-obese NAFLD had a WHtR ⩾ 0.5.

Dietary assessment

The creation and validation of DII has been documented elsewhere [18, 37]. DII was computed using 45 dietary factors collected from 11 different communities globally. A Z-score was calculated by normalizing each parameter's value through the removal of the database mean and division by the parameter's standard deviation. Then, by doubling and removing one (from -1 to +1 and centering on 0), the Z-score was transformed into percentile values [18, 38]. Each central percentile was then multiplied by its associated inflammatory impact score. Then, the DII scores for each dietary parameter were added to calculate an individual's DII.

Dietary information in NHANSE was collected via a 24-hour recall interview done at the mobile examination center (MEC). In this research, a total of 28 different food parameters, including dietary calorie intake, protein, carbohydrates, cholesterol, fat, fatty acids (saturated, monounsaturated and polyunsaturated), folic acid, beta-carotene, ω-3 and ω-6 fatty acids, niacin, fiber, alcohol, caffeine, and various vitamins (A, B1, B2, B6, B12, C, D, E), iron, zinc, selenium, and magnesium were employed in the calculation of DII, consistent with prior studies [39-41]. After gathering the data, the DII for each parameter was computed using the following formula:

$$DII_{each\_parameter} = \left(\frac{Individual's\ \text{intake}_{each\_parameter} - Global\ \text{daily mean intake}_{each\_parameter}}{the\ \text{Standard deviation of global daily mean intake}_{each\_parameter}}\right) \times the\ \text{inf}\ lammatory\ index_{each\_parameter}$$

Then, the DII of each parameter was added together to calculate a participant's overall DII. After calculating total DII, subjects were divided into quartiles: Q1: -5.20 < DII < -0.09, Q2: -0.09 ≤ DII < 1.54, Q3: 1.54 ≤ DII < 2.90, and Q4: 2.90 ≤ DII < 5.52 for

further analyses.

Covariates

Demographic data, including age, gender, race, smoking habits, family income-to-poverty ratio (PIR), educational level, together with laboratory examinations such as cholesterol (TC), TG, albumin (ALB), ALT, AST, GGT, high-density lipoprotein (HDL), PLT, and other metabolic diseases including hypertension and diabetes constituted the main covariates in this study. Hypertension was characterized as: (a) a history of hypertension, or (b) systolic blood pressure (SBP) ≥ 140 mmhg, or (c) diastolic blood pressure (DBP) ≥ 90 mmhg. Diabetes was diagnosed when: (a) a prior diagnosis of diabetes, (b) a hemoglobin A1C concentration (HbA1c) above 6.4%, or (c) a fasting plasma glucose level (FPG) over 125 mg/dL, or (d) the use of insulin.

Statistical analysis

The statistical analysis in this study were conducted using R (4.3.1). Descriptive statistics were presented in various formats, including medians, averages, standard deviations, percentages and frequencies, depending on the data attributes. In table 1 and 2, the Chi-Square Test was utilized to examine the qualitative characteristics, while One-Way ANOVA and the Kruskal-Wallis H Test were performed to compare data from groups with normal or non-normal distributions, separately. Then, three logistic models were employed to calculate odds ratios (OR) and 95% confidence intervals (CI). Model 1 represented the original model with no confounding factors adjusted. Model 2 accounted for the impacts of age, gender and races while Model 3 refined the education level, ratio of family income to poverty (PIR), hypertension,

diabetes and smoking habits based on Model 2. Subgroup analyses of age, gender, hypertension and diabetes were undertaken to assess the link between DII and NAFLD in diverse populations. All *P-values* were calculated on both sides and deemed statistically significant when below 0.05.

Results

The features of participants.

Table 1 presents the baseline characteristics of the 5,152 individuals grouped by DII quartiles. Among them, 1,289 individuals were assigned to Group Q1 (-5.20 < DII < -0.09), 1,282 to Group Q2 (-0.09 ≤ DII < 1.54), 1,293 to Group Q3 (1.54 ≤ DII < 2.90), and 1,288 to Group Q4 (2.90 ≤ DII < 5.52). Significant variations were observed among the DII groups concerning gender, race, education level, PIR, smoking habits, BMI, waist circumstance, WHtR, SBP, TC, HDL, ALB, ALT, GGT, PLT, hypertension, diabetes, NAFLD as well as advanced liver fibrosis (***p<0.05***). These disparities heightened with increasing DII scores.

Table 2 provides a complete description of both lean and obese NAFLD. Among the 2503 NAFLD patients, 86 were classified as lean NAFLD while 2417 as obese NAFLD. Lean NAFLD patients tended to be of older age, with lower DBP, higher HDL and GGT levels. Additionally, they exhibited a higher percentage of diabetes (lean NAFLD vs obese NAFLD=48.80% vs 36.80%), and a lower percentage of advanced liver fibrosis (lean NAFLD vs obese NAFLD=9.50% vs 26.80%). Moreover, noticeable racial differences could be observed between lean and obese NAFLD. Non-Hispanic Asians exhibited a higher risk of having lean NAFLD,

whereas Non-Hispanic Whites were more likely to suffer from obese NAFLD.

DII levels and NAFLD.

Table 3 displays the detailed information on the link between DII and NAFLD in multivariable logistic regression models, as previously discussed. NAFLD correlates positively with higher DII in all three models. The ORs of Q4 are 1.80 [1.54,2.10], 1.86 [1.57,2.19], 1.81 [1.48, 2.21], in the Model 1, Model 2, Model 3 separately. All *P-values* were below 0.05.

Using the same method, we evaluated the correlation between DII and advanced liver fibrosis. Overall, a notable negative relationship was observed between higher DII and advanced liver fibrosis in all three models, especially in the highest DII group (Model 1: OR=1.67 [1.28,2.17]; Model 2: OR=1.73 [1.28, 2.34]; Model 3: OR=1.46 [1.02,2.07]).

Subgroup analysis.

Table 4 summarizes the findings of the subgroup analysis. Higher DII was related with an increase likelihood of NAFLD in adults both below and above the age of 60 (Q4: age≤60, OR=1.80 [1.39,2.33];age>60, OR=1.88 [1.37, 2.60]). Generally, a higher DII elevated the risk of NAFLD in both genders, particularly in females (in group Q4: male: OR= 1.35 [1.02,1.80], female: 2.35 [1.74,3.17]). Therefore, our findings imply that women may be more vulnerable to dietary inflammation than men. Surprisingly, our findings revealed that among those without hypertension or diabetes, DII appeared to be more favorably related with risk of NAFLD.

The link between DII and lean/abdominal lean NAFLD.

Table 5 contains comprehensive information regarding the relationship between DII and lean/abdominal lean NAFLD across three logistic regression models. Model 1 remained unaltered, while Model 2 was modified for age and gender. Model 3 incorporated corrections for educational level, PIR, smoking habits, hypertension and diabetes based on the adjustments made in Model 2. Obviously, no statistical association could be observed between DII and lean/abdominal lean NAFLDs. However, a favorable correlation was identified between DII and obese/abdominal obese NAFLD (For BMI ≥25.00kg/mˆ2, Q4: model 3: OR= 1.56 [1.23,1.98]. For WHtR⩾0.5, Q4: model 3: OR=1.48 [1.23,1.79]).

Discussion

Plenty of studies have proven the negative impact of pro-inflammatory diets on metabolic diseases, including hypertension [42], heart failure [43], cognitive impairment [39] and diabetes [41]. More importantly, dietary inflammation has been observed to be essential for the development of fatty liver disease [22, 44]. However, further investigation into the relationship of DII and NAFLD is warranted. On the one hand, excessive alcohol consumption should be excluded as it serves as both a significant contributor to dietary inflammation and another leading cause of fatty liver disease. Neither Ting Tian [22] nor Mohsen Mazidi [44] were able to definitively rule out the direct effect of excessive alcohol intake in their studies, due to their emphasis on a spectrum of fatty liver conditions. On the other hand, two subtypes of lean NAFLD have been identified, recently. Type 1 is more common in those with

abdominal obesity and insulin resistance (IR), while type 2 is more commonly observed in those with monogenic disorders [45, 46]. While adopting healthy eating habits is generally recommended for all forms of NAFLDs [47], clinicians encounter challenges in selecting appropriate clinical assessments for patients with varied weights and body types. Yet limited research has explored the effect of diet on NAFLD in individuals with varying body shapes. This knowledge gap served as inspiration for our study.

The main conclusions of our investigation are as follow: Firstly, individuals adhering to a pro-inflammatory diet are more prone to NAFLD and severe liver fibrosis. Secondly, higher DII scores correlate with elevated BMI and WHtR. Moreover, the impact of dietary inflammation appears less pronounced in lean NAFLD compared to obese NAFLD. Finally, subgroup analysis indicates that female participants, and those without diabetes are particularly vulnerable to developing NAFLD when consuming a pro-inflammatory diet. The findings are consistent with earlier research revealing the effect of diet on chronic inflammation induction [13, 22, 48] and its role in NAFLD [49].

In general, diets with higher DII are associated with processed foods containing increased calories, fat, cholesterol and carbs. More importantly, the accumulation of subcutaneous and visceral fat is often observed in conjunction with poor dietary habits [40]. Initially, a pro-inflammatory diet stimulates adipose tissue to produce pro-inflammatory adipokines and cytokines, including TNF-α, IL-1, IL-6, etc.[50, 51]. These compounds cause persistent low-grade inflammation, a common etiology in

both obesity and NAFLD. Subsequently, these pro-inflammatory mediators increase the production of reactive oxygen and nitrogen species [52], as well as induce immunological dysfunction by altering macrophages [53, 54], exacerbating liver damage. Moreover, high DII diets have been linked to insulin resistance (IR) [41, 55] and the modification of gut flora [56]. Additionally, liver tissue exposure to prolonged free fatty acids (FFA) [57], one of the primary causes of NAFLD [58], is more common in obese people. Thus, we suppose obesity, and particularly abdominal obesity, mediates the development of NAFLD driven by pro-inflammatory diets. Conversely, dietary factors seem to have a lesser impact on lean NAFLD. We hypothesize the importance of genetic and epigenetic factors in the onset and progression of lean NAFLD. Previous studies supported our assumption, demonstrating that certain genetic variations, such as the G variation in PNPLA3 and the T variant in TM6SF2 are more prevalent among lean NAFLD patients [59], potentially impacting genes associated to inflammation, oxidative stress and lipid metabolism [60-62]. In a word, obese individuals may benefit more from modifying their dietary habits to prevent NAFLD, whereas lean people may require more targeted pharmacological therapies focusing on genes and downstream pathways rather than relying solely on dietary interventions. These therapies may include the use of certain anti-sense oligonucleotides, RNA interference, and medicines regulating gut flora [63]. It is encouraging that various drugs aimed at these processes are currently undergoing clinical trials, including traditional Chinese therapies such as Huazhi Fugan Granules [64], Fufang Zhenzhu Tiaozhi formula (FTZ) [65] and

Chaihu-Shugan-San, Shen-Ling-Bai-Zhu-San [66].

In subgroup analyses, women were observed to have higher DII diets and be more vulnerable to the adverse effects of DII. This gender-related difference may be attributed to variations in dietary patterns and food choices. Besides, women are more likely to weight gain, especially around menopause, when estrogen levels decline, leading to increased fat storage [67]. Also, research suggests that females are less likely to be physically active and are more prone to overeat owing to lives and emotional factors [68, 69], highlighting the necessity of optimizing diet structure in women. Surprisingly, in this study, DII exhibits a stronger favorable correlation with NAFLD among participants without hypertension or diabetes. This phenomenon persists even after re-testing. One possible explanation is that those with hypertension and diabetes are already metabolically impaired, displaying decreased insulin sensitivity. Consequently, modifying the existing metabolic dysfunction remains challenging even with dietary improvements. Moreover, hypertension and diabetes serve as both causes and significant consequences of NAFLD, potentially leading to collinearity issues in data processing. This may also be due to predisposition and other lifestyle variables, such as varying levels of activity or quality of sleep and so on. To sum up, even individuals who have not been diagnosed with hypertension or diabetes should adopt a healthy diet pattern. This not only reduces the chance of acquiring hypertension and diabetes, but also mitigates the possibility of NAFLD.

Strengths and limitations

A major highlight of this study is its wide scope and thorough preparation,

conducted within an organized multistage and cross-sectional project supervised by the NCHS. Furthermore, the program's broad inclusiveness, which includes racial diversity, ensures a robust and representative sample, thereby enhancing the dependability and quality of our research. However, some restrictions should be acknowledged. First, diagnostic uncertainty might arise owing to lack of a clear strategy or imaging data in detecting NAFLD and advanced liver fibrosis. Second, the use of questionnaires to collect data on dietary components may introduce recollection bias. Finally, despite our best efforts to adjusting for confounding variables, the potential influence of certain macronutrient-related confounders cannot be entirely avoided. However, given our attempts to reduce the impact of extraneous variables, our current findings remain valid and hold significant importance in a clinical context.

Conclusion

To sum up, this study demonstrated a notable positive connection between DII and NAFLD, as well as its' progression to advanced liver fibrosis. Significantly, the impact of dietary inflammation on NAFLD is more pronounced in obese individuals compared to their lean counterparts. Furthermore, female participants, and those without a diagnose of hypertension and diabetes appear to be more vulnerable to the negative effects of a pro-inflammatory diet. The clinical significance or our study is multifaced: Firstly, obese persons may derive greater benefits from adopting healthier eating patterns. Conversely, lean individuals may require more targeted pharmacological therapies on genes and their downstream pathways in further studies.

Secondly, the study highlights the importance of tailored dietary recommendations for specific demographic groups. Female participants and those without diagnosed hypertension or diabetes should follow a more stringent healthy eating pattern.

Acknowledgment

We appreciate the efforts of the NHANES 2003-2018 individuals in data collecting and sharing.

Reference

1. Hardy, T., et al., Nonalcoholic Fatty Liver Disease: Pathogenesis and Disease Spectrum. Annual Review of Pathology: Mechanisms of Disease, 2016. **11**(1): p. 451-496.

2. Buzzetti, E., M. Pinzani, and E.A. Tsochatzis, The multiple-hit pathogenesis of non-alcoholic fatty liver disease (NAFLD). Metabolism, 2016. **65**(8): p. 1038-1048.

3. Powell, E.E., V.W.-S. Wong, and M. Rinella, Non-alcoholic fatty liver disease. The Lancet, 2021. **397**(10290): p. 2212-2224.

4. Ahadi, M., et al., A review of non-alcoholic fatty liver disease in non-obese and lean individuals. Journal of Gastroenterology and Hepatology, 2020. **36**(6): p. 1497-1507.

5. Yip, T.C.F., et al., Geographical similarity and differences in the burden and genetic predisposition of NAFLD. Hepatology, 2023. **77**(4): p. 1404-1427.

6. Ye, Q., et al., Global prevalence, incidence, and outcomes of non-obese or lean non-alcoholic fatty liver disease: a systematic review and meta-analysis. Lancet Gastroenterol Hepatol, 2020. **5**(8): p. 739-752.

7. Dao, A.D., et al., Prevalence, characteristics, and mortality outcomes of obese and nonobese MAFLD in the United States. Hepatol Int, 2023. **17**(1): p. 225-236.

8. Eslam, M., et al., Metabolic (dysfunction)-associated fatty liver disease in individuals of normal weight. Nature Reviews Gastroenterology & Hepatology, 2022. **19**(10): p. 638-651.

9. Jang, H. and W. Kim, Non-obese or lean nonalcoholic fatty liver disease matters, but is it preventable or inevitable in light of its risk factors? Clin Mol Hepatol, 2023. **29**(2): p. 381-383.

10. Arrese, M., et al., Innate Immunity and Inflammation in NAFLD/NASH. Dig Dis Sci, 2016. **61**(5): p. 1294-303.

11. Petrescu, M., et al., Chronic Inflammation-A Link between Nonalcoholic Fatty Liver Disease (NAFLD) and Dysfunctional Adipose Tissue. Medicina (Kaunas), 2022. **58**(5).

12. Xiong, P., et al., Metaflammation in glucolipid metabolic disorders: Pathogenesis and treatment. Biomed Pharmacother, 2023. **161**: p. 114545.

13. Christ, A., M. Lauterbach, and E. Latz, Western Diet and the Immune System: An Inflammatory Connection. Immunity, 2019. **51**(5): p. 794-811.

14. Malesza, I.J., et al., High-Fat, Western-Style Diet, Systemic Inflammation, and Gut Microbiota: A Narrative Review. Cells, 2021. **10**(11).

15. Casas, R., et al., Nutrition and Cardiovascular Health. Int J Mol Sci, 2018. **19**(12).

16. Thoma, C., C.P. Day, and M.I. Trenell, Lifestyle interventions for the treatment of non-alcoholic fatty liver disease in adults: a systematic review. J Hepatol, 2012. **56**(1): p. 255-66.

17. Ryan, M.C., et al., The Mediterranean diet improves hepatic steatosis and insulin sensitivity in individuals with non-alcoholic fatty liver disease. J Hepatol, 2013. **59**(1): p. 138-43.

18. Shivappa, N., et al., Designing and developing a literature-derived, population-based dietary inflammatory index. Public Health Nutr, 2014. **17**(8): p. 1689-96.

19. Kenđel Jovanović, G., et al., The Efficacy of an Energy-Restricted Anti-Inflammatory Diet for the Management of Obesity in Younger Adults. Nutrients, 2020. **12**(11).

20. Tan, J., et al., A Proinflammatory Diet May Increase Mortality Risk in Patients with Diabetes Mellitus. Nutrients, 2022. **14**(10).

21. Zhou, N., et al., The dietary inflammatory index and its association with the prevalence of hypertension: A cross-sectional study. Front Immunol, 2022. **13**: p. 1097228.

22. Tian, T., et al., Dietary Quality and Relationships with Metabolic Dysfunction-Associated Fatty Liver Disease (MAFLD) among United States Adults, Results from NHANES 2017-2018. Nutrients, 2022. **14**(21).

23. Younes, R., et al., Long-term outcomes and predictive ability of non-invasive scoring systems in patients with non-alcoholic fatty liver disease. Journal of Hepatology, 2021. **75**(4): p. 786-794.

24. Stern, C. and L. Castera, Non-invasive diagnosis of hepatic steatosis. Hepatology International, 2016. **11**(1): p. 70-78.

25. Sourianarayanane, A. and A.J. McCullough, Accuracy of steatosis and fibrosis NAFLD scores in relation to vibration controlled transient elastography: An NHANES analysis. Clinics and Research in Hepatology and Gastroenterology, 2022. **46**(7).

26. Sharma, S., Non-invasive diagnosis of advanced fibrosis and cirrhosis. World Journal of Gastroenterology, 2014. **20**(45).

27. Ruhl, C.E. and J.E. Everhart, Fatty liver indices in the multiethnic United States

National Health and Nutrition Examination Survey. Alimentary Pharmacology & Therapeutics, 2015. **41**(1): p. 65-76.

28. Cheah, M.C.C., A.J. McCullough, and G.B.-B. Goh, Current Modalities of Fibrosis Assessment in Non-alcoholic Fatty Liver Disease. Journal of Clinical and Translational Hepatology, 2017. **XX**(XX): p. 1-11.

29. Castera, L., M. Friedrich-Rust, and R. Loomba, Noninvasive Assessment of Liver Disease in Patients With Nonalcoholic Fatty Liver Disease. Gastroenterology, 2019. **156**(5): p. 1264-1281 e4.

30. Ahluwalia, N., et al., Update on NHANES Dietary Data: Focus on Collection, Release, Analytical Considerations, and Uses to Inform Public Policy. Adv Nutr, 2016. **7**(1): p. 121-34.

31. Bedogni, G., et al., The Fatty Liver Index: a simple and accurate predictor of hepatic steatosis in the general population. BMC Gastroenterol, 2006. **6**: p. 33.

32. Long, M.T., M. Noureddin, and J.K. Lim, AGA Clinical Practice Update: Diagnosis and Management of Nonalcoholic Fatty Liver Disease in Lean Individuals: Expert Review. Gastroenterology, 2022. **163**(3): p. 764-774.e1.

33. Kim, D. and W.R. Kim, Nonobese Fatty Liver Disease. Clinical Gastroenterology and Hepatology, 2017. **15**(4): p. 474-485.

34. Rico-Martín, S., et al., Effectiveness of body roundness index in predicting metabolic syndrome: A systematic review and meta-analysis. Obes Rev, 2020. **21**(7): p. e13023.

35. Christakoudi, S., et al., Associations of body shape index (ABSI) and hip index with liver, metabolic, and inflammatory biomarkers in the UK Biobank cohort. Sci Rep, 2022. **12**(1): p. 8812.

36. Browning, L.M., S.D. Hsieh, and M. Ashwell, A systematic review of waist-to-height ratio as a screening tool for the prediction of cardiovascular disease and diabetes: 0.5 could be a suitable global boundary value. Nutr Res Rev, 2010. **23**(2): p. 247-69.

37. Wang, T., et al., The association between Dietary Inflammatory Index and disability in older adults. Clinical Nutrition, 2021. **40**(4): p. 2285-2292.

38. Darbandi, M., et al., Anti-inflammatory diet consumption reduced fatty liver indices. Sci Rep, 2021. **11**(1): p. 22601.

39. Dong, X., et al., Association of dietary omega-3 and omega-6 fatty acids intake with cognitive performance in older adults: National Health and nutrition examination Survey (NHANES) 2011-2014. Nutr J, 2020. **19**(1): p. 25.

40. Tu, J., et al., Dietary inflammatory potential is associated with sarcopenia in patients with hypertension: national health and nutrition examination study. Front Nutr, 2023. **10**: p.

1176607.

41. Shu, Y., et al., Associations of Dietary Inflammatory Index With Prediabetes and Insulin Resistance. Front Endocrinol (Lausanne), 2022. **13**: p. 820932.

42. Wu, W., et al., Association between dietary inflammation index and hypertension in participants with different degrees of liver steatosis. Ann Med, 2023. **55**(1): p. 2195203.

43. Liu, Z., et al., Association Between Dietary Inflammatory Index and Heart Failure: Results From NHANES (1999-2018). Front Cardiovasc Med, 2021. **8**: p. 702489.

44. Mazidi, M., et al., Diet with greater inflammatory potential is associated with higher prevalence of fatty liver among US adults. European Journal of Clinical Nutrition, 2019. **73**(12): p. 1653-1656.

45. Vilarinho, S., et al., Emerging Role of Genomic Analysis in Clinical Evaluation of Lean Individuals With NAFLD. Hepatology, 2021. **74**(4): p. 2241-2250.

46. Orkin, S., et al., Body composition measured by bioelectrical impedance analysis is a viable alternative to magnetic resonance imaging in children with nonalcoholic fatty liver disease. JPEN J Parenter Enteral Nutr, 2022. **46**(2): p. 378-384.

47. Pouwels, S., et al., Non-alcoholic fatty liver disease (NAFLD): a review of pathophysiology, clinical management and effects of weight loss. BMC Endocr Disord, 2022. **22**(1): p. 63.

48. Barbaresko, J., et al., Dietary pattern analysis and biomarkers of low-grade inflammation: a systematic literature review. Nutrition Reviews, 2013. **71**(8): p. 511-527.

49. Schuster, S., et al., Triggering and resolution of inflammation in NASH. Nature Reviews Gastroenterology & Hepatology, 2018. **15**(6): p. 349-364.

50. Wang, T. and C. He, Pro-inflammatory cytokines: The link between obesity and osteoarthritis. Cytokine Growth Factor Rev, 2018. **44**: p. 38-50.

51. Kawai, T., M.V. Autieri, and R. Scalia, Adipose tissue inflammation and metabolic dysfunction in obesity. American Journal of Physiology-Cell Physiology, 2021. **320**(3): p. C375-C391.

52. Abdollahzad, H., et al., The Relationship Between the Dietary Inflammatory Index and Metabolic Syndrome in Ravansar Cohort Study. Diabetes Metab Syndr Obes, 2020. **13**: p. 477-487.

53. Kapellos, T.S., et al., Human Monocyte Subsets and Phenotypes in Major Chronic Inflammatory Diseases. Front Immunol, 2019. **10**: p. 2035.

54. Figueroa-Vega, N., et al., Analysis of the percentages of monocyte subsets and ILC2s, their relationships with metabolic variables and response to hypocaloric restriction in obesity. PLoS One, 2020. **15**(2): p. e0228637.

55. Martins, F.O. and S.V. Conde, Impact of Diet Composition on Insulin Resistance. Nutrients, 2022. **14**(18).

56. Lau, L.H.S. and S.H. Wong, Microbiota, Obesity and NAFLD. Adv Exp Med Biol, 2018. **1061**: p. 111-125.

57. Dietrich, P. and C. Hellerbrand, Non-alcoholic fatty liver disease, obesity and the metabolic syndrome. Best Practice & Research Clinical Gastroenterology, 2014. **28**(4): p. 637-653.

58. Polyzos, S.A., J. Kountouras, and C.S. Mantzoros, Adipose tissue, obesity and non-alcoholic fatty liver disease. Minerva Endocrinol, 2017. **42**(2): p. 92-108.

59. Feldman, A., et al., Clinical and Metabolic Characterization of Lean Caucasian Subjects With Non-alcoholic Fatty Liver. American Journal of Gastroenterology, 2017. **112**(1): p. 102-110.

60. Aggeletopoulou, I., et al., Epigenetic Regulation in Lean Nonalcoholic Fatty Liver Disease. International Journal of Molecular Sciences, 2023. **24**(16).

61. Shen, J., et al., PNPLA3 gene polymorphism and response to lifestyle modification in patients with nonalcoholic fatty liver disease. Journal of Gastroenterology and Hepatology, 2014. **30**(1): p. 139-146.

62. Wong, V.W.-S., et al., Incidence of non-alcoholic fatty liver disease in Hong Kong: A population study with paired proton-magnetic resonance spectroscopy. Journal of Hepatology, 2015. **62**(1): p. 182-189.

63. Bluemel, S., et al., Precision medicine in alcoholic and nonalcoholic fatty liver disease via modulating the gut microbiota. Am J Physiol Gastrointest Liver Physiol, 2016. **311**(6): p. G1018-G1036.

64. Ye, M., et al., Alleviation of non-alcoholic fatty liver disease by Huazhi Fugan Granules is associated with suppression of TLR4/NF-κB signaling pathway. Clínica e Investigación en Arteriosclerosis (English Edition), 2021. **33**(5): p. 257-266.

65. Wang, H., et al., FTZ attenuates liver steatosis and fibrosis in the minipigs with type 2 diabetes by regulating the AMPK signaling pathway. Biomedicine & Pharmacotherapy, 2021. **138**.

66. Yang, Q.-H., et al., Effects of Chaihu-Shugan-San and Shen-Ling-Bai-Zhu-San on p38 MAPK Pathway in Kupffer Cells of Nonalcoholic Steatohepatitis. Evidence-Based Complementary and Alternative Medicine, 2014. **2014**: p. 1-8.

67. Zhao, Q., et al., The Relationship between the Dietary Inflammatory Index (DII) and Metabolic Syndrome (MetS) in Middle-Aged and Elderly Individuals in the United States. Nutrients, 2023. **15**(8).

68. Sokol, A., et al., Association between the dietary inflammatory index, waist-to-hip ratio and metabolic syndrome. Nutr Res, 2016. **36**(11): p. 1298-1303.

69. Meyer, M.L., et al., Polycystic Ovary Syndrome Signs and Metabolic Syndrome in Premenopausal Hispanic/Latina Women: the HCHS/SOL Study. J Clin Endocrinol Metab, 2020. **105**(3): p. e447-56.

202402211800369577406733330475008

ORIGINALITY REPORT

14%

SIMILARITY INDEX

PRIMARY SOURCES

1	www.mdpi.com<br>Internet	54 words — 1%
2	Lurao Li, Yun Yi, Xiawen Shu, Jianghui Li, Hui Kang, Ying Chang. "The Correlation Between Serum Copper and Non-alcoholic Fatty Liver Disease in American Adults: an Analysis Based on NHANES 2011 to 2016", Biological Trace Element Research, 2024<br>Crossref	51 words — 1%
3	www.researchgate.net<br>Internet	20 words — < 1%
4	bmcendocrdisord.biomedcentral.com<br>Internet	19 words — < 1%
5	assets.researchsquare.com<br>Internet	16 words — < 1%
6	"Steatohepatitis Clinical Themes", Hepatology, 2013<br>Crossref	15 words — < 1%
7	lipidworld.biomedcentral.com<br>Internet	14 words — < 1%
8	pjms.org.pk<br>Internet	14 words — < 1%

9	www.acc.org<br>Internet	14 words — < 1%
10	pubmed.ncbi.nlm.nih.gov<br>Internet	13 words — < 1%
11	www.nature.com<br>Internet	13 words — < 1%
12	Rohit Hariharan, Emmanuel Nene Odjidja, David Scott, Nitin Shivappa, James R. Hébert, Allison Hodge, Barbora Courten. "The dietary inflammatory index, obesity, type 2 diabetes, and cardiovascular risk factors and diseases", Obesity Reviews, 2021<br>Crossref	12 words — < 1%
13	bmcpublichealth.biomedcentral.com<br>Internet	12 words — < 1%
14	link.springer.com<br>Internet	12 words — < 1%
15	www.frontiersin.org<br>Internet	12 words — < 1%
16	Ana M. López-Sobaler, Aránzazu Aparicio, Javier Aranceta-Bartrina, Ángel Gil et al. "Overweight and General and Abdominal Obesity in a Representative Sample of Spanish Adults: Findings from the ANIBES Study", BioMed Research International, 2016<br>Crossref	11 words — < 1%
17	article.imrpress.com<br>Internet	11 words — < 1%
18	journals.plos.org<br>Internet	11 words — < 1%

19	www.ejh.it<br>Internet	11 words — < 1%
20	www.researchsquare.com<br>Internet	11 words — < 1%
21	www.tandfonline.com<br>Internet	11 words — < 1%
22	Pierre Bedossa, Rami Moucari, Emna Chelbi, Tarik Asselah et al. "Evidence for a role of nonalcoholic steatohepatitis in hepatitis C: A prospective study", Hepatology, 2007<br>Crossref	10 words — < 1%
23	ijponline.biomedcentral.com<br>Internet	10 words — < 1%
24	Azam Doustmohammadian, Bahareh Amirkalali, Saeed Esfandyari, Nima Motamed et al. "The association between dietary inflammatory index (DII) scores and c-reactive protein (CRP) and nonalcoholic fatty liver disease (NAFLD) in a general population cohort", Clinical Nutrition ESPEN, 2024<br>Crossref	9 words — < 1%
25	Maryam Taghdir, Akram Salehi, Karim Parastouei, Sepideh Abbaszadeh. "Relationship between diet quality and nonalcoholic fatty liver disease predictor indices in Iranian patients with metabolic syndrome: A cross-sectional study", Food Science & Nutrition, 2023<br>Crossref	9 words — < 1%
26	Ramy Younes, Elisabetta Bugianesi. "NASH in Lean Individuals", Seminars in Liver Disease, 2019<br>Crossref	9 words — < 1%

27 Weihua Dong, Man Qing-qing, Zhang Jian, Liu Zhen, Gong Wei-yi, Zhao Li-yun, SONG Peng-kun, Ding Gang-qiang. "Association between the Dietary Inflammatory Index and Hypertension in Middle-Aged and Elderly population in China: Results from a nation-wide cross sectional study", Research Square Platform LLC, 2023 — 9 words — < 1%
Crossref Posted Content

28 covid19dataportal.es — 9 words — < 1%
Internet

29 www.gavinpublishers.com — 9 words — < 1%
Internet

30 www.wjgnet.com — 9 words — < 1%
Internet

31 www.xiahepublishing.com — 9 words — < 1%
Internet

32 Ho Soo Chun, Minjong Lee, Jae Seung Lee, Hye Won Lee et al. "Metabolic dysfunction associated fatty liver disease identifies subjects with cardiovascular risk better than nonalcoholic fatty liver disease", Liver International, 2022 — 8 words — < 1%
Crossref

33 Na Wu, Xiangyu Zhai, Fan Yuan, Jie Li, Dong Li, Jianying Wang, Lei Zhang, Yi Shi, Guang Ji, Guang He, Baocheng Liu. "Genetic variation in TBC1 domain family member 1 gene associates with the risk of lean NAFLD via high-density lipoprotein", Frontiers in Genetics, 2023 — 8 words — < 1%
Crossref

34 Qianwen Zhao, Yunlei Deng, Rong Gong, Ting Chen, Li Yang. "Association between dietary — 8 words — < 1%

inflammatory index and risk of fatty liver disease: A systematic review and meta-analysis", Digestive and Liver Disease, 2023
Crossref

35 Thanita Thongtan, Anasua Deb, Wasawat Vutthikraivit, Passisd Laoveeravat, Thammasak Mingbunjerdsuk, Sameer Islam, Ebtesam Islam. "Antiplatelet therapy associated with lower prevalence of advanced liver fibrosis in non-alcoholic fatty liver disease: A systematic review and meta-analysis", Indian Journal of Gastroenterology, 2022 — 8 words — < 1%
Crossref

36 Wei Li, Haitao Xiao, Hong Wu, Xuewen Xu, Yange Zhang. "Organophosphate pesticide exposure and biomarkers of liver injury/liver function", Liver International, 2022 — 8 words — < 1%
Crossref

37 bmcgeriatr.biomedcentral.com — 8 words — < 1%
Internet

38 journals.lww.com — 8 words — < 1%
Internet

39 worldwidescience.org — 8 words — < 1%
Internet

40 www.tara.tcd.ie — 8 words — < 1%
Internet

41 "Metabolic Syndrome", Springer Science and Business Media LLC, 2023 — 7 words — < 1%
Crossref

42 Mohammad Shafi Kuchay, José Ignacio Martínez-Montoro, Narendra Singh Choudhary, José Carlos Fernández-García et al. "Non-Alcoholic Fatty Liver Disease in — 7 words — < 1%

Lean and Non-Obese Individuals: Current and Future Challenges", Biomedicines, 2021
Crossref

43 "55th EASD Annual Meeting of the European Association for the Study of Diabetes", Diabetologia, 2019 — 6 words — < 1%
Crossref

44 Achuthan Sourianarayanane, Arthur J McCullough. "Accuracy of Steatosis and Fibrosis NAFLD scores in relation to Vibration Controlled Transient Elastography: an NHANES analysis", Clinics and Research in Hepatology and Gastroenterology, 2022 — 6 words — < 1%
Crossref

45 Harry Muhammad, Marleen van Baak, Edwin Mariman, Dian Sulistyoningrum, Emy Huriyati, Yi Lee, Wan Wan Muda. "Dietary Inflammatory Index Score and Its Association with Body Weight, Blood Pressure, Lipid Profile, and Leptin in Indonesian Adults", Nutrients, 2019 — 6 words — < 1%
Crossref

46 Lee, Yong-ho, Kyu Sik Jung, Seung Up Kim, Hye-jin Yoon, Yu Jung Yun, Byung-Wan Lee, Eun Seok Kang, Kwang-Hyub Han, Hyun Chul Lee, and Bong-Soo Cha. "Sarcopaenia is associated with NAFLD independently of obesity and insulin resistance: Nationwide surveys (KNHANES 2008–2011)", Journal of Hepatology, 2015. — 6 words — < 1%
Crossref

47 Leila Nikniaz, Zeinab Nikniaz, Nitin Shivappa, James R. Hébert. "The association between dietary inflammatory index and metabolic syndrome components in Iranian adults", Primary Care Diabetes, 2018 — 6 words — < 1%
Crossref

48	Mingkun Yang, Shenhui Miao, Weihang Hu, Jing Yan. "Association between the dietary inflammatory index and all-cause and cardiovascular mortality in patients with atherosclerotic cardiovascular disease", Nutrition, Metabolism and Cardiovascular Diseases, 2023<br>Crossref	6 words — < 1%
49	Nawoda Hewage, Udaya Wijesekara, Rasika Perera. "Determining the best method for evaluating obesity and the risk for non-communicable diseases in women of childbearing age by measuring the body mass index, waist circumference, waist-to-hip ratio, waist-to-height ratio, A Body Shape Index, and hip index", Nutrition, 2023<br>Crossref	6 words — < 1%
50	Rachel S. Bergmans, Kristen M. Malecki. "The association of dietary inflammatory potential with depression and mental well-being among U.S. adults", Preventive Medicine, 2017<br>Crossref	6 words — < 1%

EXCLUDE QUOTES	OFF	EXCLUDE SOURCES	OFF
EXCLUDE BIBLIOGRAPHY	ON	EXCLUDE MATCHES	OFF
